# Supplementary material for: T-Cadherin Finetunes Proliferation–Differentiation During Adipogenesis via PI3K–AKT Signaling Pathway
Source: Int J Mol Sci. 2025 Oct 2;26(19):9646. doi: 10.3390/ijms26199646 (PMC12524427; doi:10.3390/ijms26199646)
Supplement: Supplementary file 1 [file ijms-26-09646-s001.zip › ijms-3869874-supplementary.pdf]

Supplementary table S1: Primer sequence details.

Gene	Primer sequence	
	Forward (5’-3’)	Reverse (5’-3’)
AdipoQ	CATGCCGAAGATGACGTTAC	AGGACCAAGAAGACCTGCAT
CDH13 108	GCCCTCGTGAGCCTTCTTC	CACCCTGAGGTCCGTGATGT
PPAR-γ	TGAAAGAAGCGGTGAACCACTG	TGGCATCTCTGTGTCAACCATG
PLIN1	GCTGTCTGAGACTGAGGTGG	TGCAGAACTCTCTGGAGCAC
Leptin	GAAAATGTGCTGGAGACCCC	TCCAAGCCAGTGAGCCTCT
mTOR	TTCCTGAACAGCGAGCACAA	GTAGCGGATATCAGGGTCAGG
CD142	ACCTTACCGAGACACAAACCT	CTTTCCCGTGCTTGAGCCTT
RPLPO -13	CCCCACAAGACCAAGAGAGG	CCCCAGGTAAGCAAACTTTCTG

Supplementary table S2: Primary antibody for Immunoblot analysis.

Primary antibody	Dilution	MW	Cat. No.
**anti-H Cadherin [EPR9621]**	1/1000	105<br>130	ab167407 abcam, USA
**anti-Adiponectin**	1/1000	26	ab22554 abcam, USA
**anti-APPL1**	1/1000	79	MA5-26917 Invitrogen, USA
**anti-PI3 Kinase p110 beta**	1/1000	110	ab151549 abcam, USA
**anti-ERK1/2**	1/1000	42<br>44	ab17942 abcam, USA
**anti-phosphoERK1/2 (Erk1 (pT202/pY204) + Erk2 (pT185/pY187)**	1/1000	42<br>44	ab4819 abcam, USA
**anti-AMPKalpha**	1/1000	64	BF2001 Affinity Biosciences, China
**anti-Phospho-AMPKalpha (Thr172) (40H9)**	1/1000	62	#2535 Cell Signaling Technology, Inc., USA
**anti-Akt (5G3)**	1/1000	60	#2966 Cell Signaling Technology, Inc., USA
**anti-phospho-Akt (Ser473) (D9E) XP®**	1/1000	60	#4060 Cell Signaling Technology, Inc., USA
**anti-GAPDH**	1/5000	37	sc-32233 Santa Cruz Biotechnology, Inc., USA
**anti-actin (20-33)**	1/1000	42	A5060 SigmaAldrich, USA
**anti-H Cadherin antibody [EPR9621] ab167407**	1/500	105<br>130	Abcam
**anti-ERGIC53 rabbit polyclonal Santa Cruz sc-66880**	1/1000	53	Santa Cruz Biotechnology |
